# The link Between Stock Exchange Sectors and Indices: Implications During the COVID-19 Pandemic

**DOI:** 10.1177/21582440221142756

**Published:** 2022-12-20

**Authors:** Florin-Teodor Boldeanu, Adriana Veronica Litră, José Antonio Clemente-Almendros, Ileana Tache

**Affiliations:** 1 Transilvania University of Brasov, Romania; 2 Universidad Internacional de La Rioja, Logroño, Spain

**Keywords:** COVID-19, event study, stock market indices, sectorial analysis, lockdown, announcement

## Abstract

The paper investigates the impact of the COVID-19 pandemic, using the event study approach for the Bucharest Stock Exchange, by which Bucharest Stock Indices and listed firms grouped by sectors were analyzed. The paper uses three important event days, 20 January 2020, 11 March 2020, and 15 March 2020. The findings demonstrate that initially investors were not concerned about the pandemic, showing that they did not realize the extent of globalization and transmission of events on financial markets. Both after 11 March and after 15 March 2020, stock indices have declined, investors becoming worried about the prospects of their dividends and the stock liquidity. The most affected sectors were those related to metallurgical industry, IT&C. After the lockdown, there was a reversal for some sectors like pharmaceutical and biotechnology, electricity production, transportation and distribution, and IT&C. Understanding the intensity and direction of the link between some sectors and indices may influence investment strategies and help in hedging, especially in times of pandemics.

## Introduction

The pandemic of COVID-19 has been an event that marked the year 2020 and the social and economic landscape will probably change in the years or decades to come. Research has seen a new revival in fields like economics, medicine, psychology, or education, as the pandemic has had a big impact in different areas from online learning, mental depression, social conflicts, decline in share prices, and many more (Ding et al., 2021; Ettman et al., 2020; Narayan et al., 2021). Moreover, the different restrictions affected the mobility of people and goods and have had environmental implications, such as the improvement of the air quality (Shehzad, Bilgili, Koçak, et al., 2021). Analyzing how the different stringent policies have mitigated the spread of COVID-19 may help in future situations (Bilgili et al., 2021).

Regarding the financial market, the pandemic has caused considerable and severe declines in share prices starting in China and, as the virus spread, all around the world. After the initial outbreak in Wuhan, Hubei Province, the Chinese authorities decided to restrict travel on 20 January 2020. The economic crisis created by COVID-19 negatively affected the stability of financial markets (Shehzad, Xiaoxing, et al., 2021). This caused a negative impact on the stock market in China (He et al., 2020; Liu et al., 2020). Once the virus spread worldwide, the negative impact of the new pandemic was felt on every stock market, developed, developing, or frontier markets (Cao et al., 2020; Heyden & Heyden, 2021; Phan & Narayan, 2020; B. Singh et al., 2020; Zhang et al., 2020).

As the financial market is now very globalized, world pandemics can have a considerable influence on each type of market, but the particular impact may be different (He et al., 2020; B. Singh et al., 2020). The aim of this paper is to examine the stock exchange reaction regarding different indices from the same market, the Bucharest Stock Exchange, with constituents from different sectors. The analysis includes not only an overview of the main index in relation to an important health crisis event like COVID-19, but also the impact of other stock market indices, which can help in diversifying investors’ strategies and governments’ policies. At the same time, this paper shows the impact of the pandemic on 18 different stock sectors and analyze the positive or negative link between stock market indices and stock sectors, which can also help investors and policymakers as stated before. We contribute to the literature in different ways. Firstly, by analyzing stock market indices for an emerging market, the Bucharest Stock Exchange, we investigate if indices that measure for example financial investment companies or the energy sector in a country were less affected by the pandemic than other indices of the same market. We analyze, for example, the energy index as is important in the context related to climate change and a strategic one for many countries. The Bucharest Exchange Trading Energy & Related Utilities Index (BET-NG) fell more than the other ones after 20 January 2020, correlated to a decrease between 10% and 30% in electricity demand in Europe (IEA, 2020). For the World Health Organization (WHO) announcement and the country lockdown, the reaction was negative for the five indices. The impact for the Bucharest Exchange Trading (BET), Bucharest Exchange Trading—Investment Funds (BET-FI), Bucharest Exchange Trading Total Return Index (BET-TR), and Bucharest Exchange Trading Plus Index (BETPlus) indices was pronounced, with BET-NG (the energy index) not having the biggest fall, linked with the reversal of energy consumption after the initial fall at the beginning of the first quarter of 2020 (IEA, 2020). Secondly, another important point in the article is the use of three event days (the announcement of the virus in China, the announcement of a pandemic by the WHO, and the Romanian lockdown). To the best of our knowledge, the literature has been concentrated on a specific event day (Liu et al., 2020; Pandey & Kumari, 2021; Polemis & Soursou, 2020). Because of the rapid spread of the virus around the world, there were major events happening in a short period, for example, the Romanian lockdown was announced 4 days after the WHO declared COVID-19 a pandemic. Using multiple event days will accurately determine the impact of each of them. This is in line also with behavior financial concepts, where emergencies have an impact on investors’ behavioral and psychological factors, with a direct negative effect on stock prices. Pessimism regarding the outcome of COVID-19 will have an impact on the economic environment, determining stock price changes (Espinosa-Méndez & Arias, 2020; He et al., 2020). By this process, we have demonstrated that the major negative impact on the stock market was that of the lockdown, followed by the day when WHO declared the virus a pandemic. Investors on the Bucharest Stock Exchange were in a state of panic during the first weeks of March 2020 and calmed down after the declaration of the lockdown.

Thirdly, we investigated 56 listed companies on the Bucharest Stock Exchange, grouping them in 18 sectors related to their economic activity. As in the case of the indices, the firms were not initially affected by the events happening in China in the month of January 2020. With the virus becoming a health issue both in Europe and in Romania, the transportation, hospitality, banking and heavy industry, metallurgical industry, ITC, manufacturing of machinery and equipment sectors were the most affected in the initial phases. After the lockdown, there was a reversal for sectors like manufacture of paper and paper products, pharmaceutical and biotechnology, manufacture of electrical and optical equipment, electricity production, transportation and distribution, and information and communications. Banking, real estate, manufacture of beverages, or construction are among the sectors that were negatively affected by the lockdown after 10 days of the event. Healthcare, consumer staples, and ITC are the sectors that managed a V-turn because, first of all, the health sector was at the forefront in fighting against the pandemic. IT&C has been helped by implementing the work (and study) from home. Consumer staples were influenced by the growing demand because of the pandemic (Liu et al., 2020; Narayan et al., 2021).

The fourth and final contribution is the link between indices and sectors related to the abovementioned stock exchange. There was an important link between the energy sectors and the energy index and for the non-banking sector and the investment funds index. The transportation and storage (of fuel and other energy products) had a significant drop during the pandemic, followed by electricity production, transportation and distribution and crude oil and natural gas and services, as was the case for the energy index. The share of each sector in the overall index played an important part in the total return for each event window. The same significant link was observed for the investment funds index and non-banking sector, the trend moving in the same pattern.

The remainder of this paper is organized as follows. Section “Literature Overview” presents a brief literature overview and formulates the research hypotheses. Section “Methodology and Data” describes the methodology used and data. Section “Empirical Results” highlights and discusses the main results of the investigation. Section “Conclusion” is dedicated to the concluding remarks and implications; finally, Section “Limitations and Future Research” presents the study limitations and some lines for future research.

## Literature Overview

The literature on the impact of emergencies on stock markets is consistent and diverse. It has shown that major events, for example, natural disasters, terrorist attacks, corporate events, public news, political crises, sport events, aviation disasters, had a material impact on the stock market (Shen & Zhang, 2020; B. Singh et al., 2020).

For example, Hadi et al. (2019) investigate the effects of terrorist attacks on the stock performance of tourism, travel and leisure industries, and use the event study approach in order to examine the relationship between terrorism and tourism stock performance.

Using the dynamic panel data model (System-GMM), Rezgallah et al. (2019) study the impact of political instability risk on risk-taking in the banking sector of 75 countries, representing the first attempt for this nexus, based on international evidence.

Taking into account the risks posed by the scarcity of energy resources, other authors, like Katircioğlu et al. (2019) use alternative vector autoregressive M-GARCH models in studying the interaction between the factors (alternative energy resources) and the spill-over effect.

As in investigating the relationship between health crises and market return, applying the Fama-French model developed by Nobel prize winners Eugen Fama and his colleague Kenneth French in the 1990s, a reference should be made to another relevant study co-authored by Shaeri et al. (2016), who, analyzing the oil price risk exposure of financial and non-financial subsectors, include the oil price risk factor into the Fama and French five-factor asset pricing model and identify the structural breaks in the equity returns.

The recent pandemic has reignited the interest in researching the effect of events caused by healthcare issues and the effects on the economy. By examining how different asset returns (gold, crude oil, world equities, currencies, bonds) reacted to the shocks of the COVID-19, Bouri, Cepni, et al. (2021) concluded that there was a clear evidence for strong spillover effects in financial markets. The global financial system is unstable in relation to major shocks and provides an abrupt spike in its risk build-up due to the COVID-19 outbreak. They found that the pandemic has altered the network of connectedness across the five studied assets, by generating sudden increases in both the system overall connectedness and the cross-asset connectedness of various cases. Other two studies examining the return spillover among the US stock market sectors (Shahzad, Bouri, et al., 2021) and Chinese stock sector indices (Shahzad, Naeem, et al., 2021) indicating that the relationships between global risk aversion and the spillovers are not linear, but asymmetric, which strengthens significantly during the COVID-19 outbreak. Their findings were based on the evidence that risk aversion and sentiment dynamics are important contributing factors of risky assets like stock prices. A major event like the COVID-19 has a strong and asymmetric impact on the network of volatility spillovers among sector indices.

By examining systemic distress risk spillover between the global stock market and individual stock markets in the countries most affected by the COVID-19 pandemic, Abuzayed et al. (2021) revealed that the systemic risk contagion intensified due to this outbreak, concluding that developed stock markets in North America and Europe transmitted and received more marginal extreme risk to and from the global market than Asian stock markets. The overall results of their study showed a high degree of integration in the extreme downside risk of the stock market system during the COVID-19 outbreak period.

Bouri, Naeem, et al. (2021) examined the impact of three different policies (lockdown, a stimulus package, and a travel ban) adopted by the New Zealand government as a response to the COVID-19 outbreak on 14 industry equity returns. They found that only the lockdown has had a positive impact on aggregate stock returns, which suggested that this government initiative has increased investors’ confidence in the market, which was not noticed in the case of the other two policies (the stimulus package and the travel ban). At the industry level, the impact of the three response policies was generally positive but heterogeneous across industry stock indices.

The outbreak and spread of COVID-19 have affected economies and life in general and influenced public health and government finances, causing massive losses for different industries and affecting investor confidence (Cao et al., 2020; Narayan et al., 2021). During the pandemic, governments worldwide have tried to minimize the effects of it as much as possible (Bilgili et al., 2021). In addition, COVID-19 has proven to be a unique event for analyzing the effect of social and economic restrictions on the environment (Shehzad, Bilgili, Koçak, et al., 2021). Because the COVID-19 does not have a similar correspondent in the last decades, and only occurred since January 2020, so the time span is quite limited, classical methods of analysis are difficult to be applied for predicting the long-term impact.

The analysis of the stock market’s reaction helps to understand the influence of these types of events on the economy (Liu et al., 2020). Moreover, there was a spillover impact of the global economy deceleration on the worldwide financial volatility (Shehzad, Xiaoxing, et al., 2021). In the same vein, understanding how the different stringency policies have helped to diminish COVID-19 total cases may be useful for future pandemics (Bilgili et al., 2021). The initial outbreak in Wuhan, Hubei Province determined the Chinese authorities to restrict travel on 20 January 2020. This caused a negative impact on the stock market in China (He et al., 2020; Liu et al., 2020). The outbreak of the virus in China did not have an immediate outcome on stock exchanges around the world, as panic did not propagate among investors. The first 10 days after the restrictions in China on 20 January 2020 saw limited drops for developed or developing stock markets indices and sectors (Kamran et al., 2020; B. Singh et al., 2020). The uncertainty created by the pandemic resulted in changes in the stock markets. However, the reactions of the stock markets, sectors, and indices have proven to be different (Clemente-Almendros et al., 2021; He et al., 2020; Shehzad, Xiaoxing, et al., 2021). The empirical evidence shows that the impact has not been equal across the sectors worldwide, since the various nature of their activities was itself affected differently. Additionally, understanding the impact of COVID-19 for stock markets indices may shed more light on the real and global effect of the pandemic, since the indices gather companies that have financial characteristics in common, which could be different to the sectorial grouping. Moreover, even though the index can contain companies that belong to the same sector, they can react differently because of the existence of sub-sectors, this being the case of energy companies, having different reactions.

This brings us to the following first hypothesis related to the indices and afterwards to the market sectors:

**Hypothesis 1a:** The announcement of a new virus in China on 20 January 2020 had a negative impact on Romanian stock market indices
**Hypothesis 1b:** The announcement of a new virus in China on 20 January 2020 had a negative impact on the Romanian stock market sectors

The declaration of a world pandemic in March 2020 and the spread of the virus across major economies has had serious implications. There were negative variations for stock market indices, and, as a result, for different sectors. Stock market indices tended to decline with COVID-19’s local spread severity (Cao et al., 2020; Just & Echaust, 2020). The pandemic generated great risk and uncertainty in the global financial markets; the great uncertainty of the pandemic, in combination with economic shortages, have caused markets to become highly volatile and unpredictable (Ji et al., 2020). We expect a negative effect of the announcement on stock indices and sectors:

**Hypothesis 2a:** The announcement of a pandemic on 11 March 2020 by the WHO had a negative impact on Romanian stock market indices
**Hypothesis 2b:** The announcement of a pandemic on 11 March 2020 by the WHO had a negative impact on the Romanian stock market sectors

By locking down economic activity and introducing travel bans, stock prices in most of the countries reacted negatively. For 75% of the countries most affected by COVID-19, some sort of travel ban had supported the recovery of financial markets; for the countries where markets did not recover, the travel ban was applied too late. In part, the announcement of lockdown had a positive response compared to the pandemic declaration on 11 March 2020 and travel bans. The market reacted positively with significant positive returns during post-lockdown period; investors anticipated the lockdown and reacted positively, whereas in the pre-lockdown period investors panicked, as reflected by negative returns (Alam et al., 2020; Phan & Narayan, 2020).

**Hypothesis 3a:** The lockdown in Romania on 15 March 2020 have a positive impact on the Romanian stock exchange indices.
**Hypothesis 3b:** The lockdown in Romania on 15 March 2020 have a positive impact on the Romanian stock exchange sectors.

Some companies are included as components of different stock indices or exchange-traded funds (e.g., CAC40 is composed of 40 listed companies or DAX has 30 listed companies that makes the index). Not all companies from a specific country are components of an index and some sector indices have only few major companies from that sector as constituents. Knowing if and in what index a company is included can be an important part in determining the future price of that companies’ stock and in case of events how it might react. News that influences markets and whole sectors may have an impact on all of the companies of the underlying index, even if the news is related or not to the individual company. The so far research about the COVID19 pandemic concentrated on demonstrating the negative impact on specific indices, like for example the Dow Jones, S&P500, Shanghai Composite Index, or the effect on sectors (e.g., mining, energy, transportation, or industry) (He et al., 2020; Heyden & Heyden, 2021; Huo & Qiu, 2020; B. Singh et al., 2020), but did not analyze the potential link between indices and sectors, as companies related to sectors are included in certain indices:

**Hypothesis 4:** There is a link between the evolution of stock exchange indices and sectors for the Bucharest Stock Exchange.

## Methodology and Data

The COVID-19 pandemic can be considered a unique event. Unprecedented events (stock splits, dividend announcements, earnings surprises) can have an important effect on financial markets (Ball & Brown, 1968; Fama et al., 1969). Using the event study method, the analysis concentrated on examining the abnormal returns of a sample of stock indices and firm stocks as a consequence of the pandemic, which has not yet concluded. The stock market that we have analyzed is the Bucharest Stock Exchange, which was classified as a frontier market until 21 September 2020, when FTSE Russell upgraded it to an emerging market.

The three main models used in an event study approach for calculating abnormal returns are the average adjusted return rate model, the market index adjusted return rate model, and the market model. The last one is widely used because of its efficiency and predictive power. The market model is used in this article for determining abnormal returns (ARs) and cumulative abnormal returns (CARs) for Romanian stock indices. For a sample of firms that are traded on the Bucharest Stock Exchange, we used the Fama and French three-factor model (Fama et al., 1993), an extension of the capital asset pricing model (CAPM) originating from the market model, which takes into account a size factor and a value factor. In an event study methodology, we have to establish some moments in time, represented by the estimation window, the event day, and the event window.

We used three event days, considered relevant during the COVID-19 pandemic and important for our hypotheses, with a link to behavior finance by the fact that we can see the evolution in time of investors’ sentiment. Using three event days can improve the analysis and the literature written so far, considering that it is important to isolate the significant moments during the COVID-19 outbreak. Because of the fast pace of the events following the propagation of the virus in Europe (the first announced cases, lockdowns, social and political measures), the event study approach should be employed for different time horizons. The event days used are: 20 January 2020 when the Chinese announced that the virus is very contagious and people must take precautions, 11 March 2020 when the WHO declared COVID-19 a pandemic and 15 March 2020, the declaration of the Romanian lockdown. Because 15 March 2020 was on a Sunday, the event day for the third event is placed on Monday 16 March 2020, a trading day. The estimation window for the indices consists of 250 trading days for the first event, 286 trading days for the second event, and 299 days for the third one, following the literature which considers having more than 200 days for robust results, in order to improve the accuracy of our results and to ensure that the results are not affected by stocks subject to long trading suspensions (Heyden & Heyden, 2021). The event window is defined as t ∈ (−10, 10). We use five different intervals, in increments of 5 days before and after the event: (−10, 0), (−5, 0), (0, 0), (0, 5), and (0, 10). The event window of 10 days was chosen to measure the impact of the event, as positive abnormal returns started 5 to 6 days after the event. Additionally, calculations were done for (0, 20), (0, 30), (0, 40), available on request, to show that the event had no long lasting (prolonged) effect (Schell et al., 2020), with results revealing that the cumulative average abnormal returns were positive after 10 to 20 days. The period for the estimation window starts on thirrd of January 2019 and ends 11 days before the event day. For the company stocks, following the research literature (Huo & Qiu, 2020), we eliminated companies with fewer than 60 trading days for the estimation window and having less than 10 trading days in the month of December 2019. From a sample of 83 firms traded on the Bucharest Stock Exchange, 56 of them follow the criteria mentioned above.

The event window considers 10 days before the event and 10 days after it, with an interval of 5 days for the cumulative abnormal returns. We have chosen a period before the event in order to capture market sentiment or panic which will be relevant around 20 January 2020 and after the propagation of the virus worldwide. The *T*-test was used to determine the significance of the results and the ordinary least square regression to determine the parameters of the following model.

The market model used for the Bucharest Stock Exchange indices is:

(1) $$R_{i,t}=\alpha_{i}+\beta_{i}R_{m,t}+\varepsilon_{i,t}$$

where *R_i_*,*t* are the returns for the Romanian stock index *i* and *R*_m,*t*_ are the returns for the market on day *t* in the estimation window for each event day and ε_i,*t*_ is the stochastic disturbance that has to satisfy the condition:

(2) $$E\left(\varepsilon_{i,t}\right)=0,\mathrm{VAR}\left(\varepsilon_{i,t}\right)=\sigma_{i},$$

After calculating the α_*i*_ and β_*i*_ using the actual returns of the index, we determine the expected return for stock index *i* on day *t* from *t*_0_ to *t*_1_:

(3) $$E\left(R_{i,t}\right)={\hat{\alpha }}_{i}+{\hat{\beta }}_{i}R_{m,t}$$

The abnormal returns (AR) for stock index *i* on day *t* during the event window *t*_0_–*t*_1_ and the cumulative abnormal returns (CAR) are as follows:

(4) $$AR_{i,t}=R_{i,t}-E\left(R_{i,t}\right)$$

(5) $$\mathrm{CA}R_{i}\left(t_{0},t_{1}\right)=\sum_{t=t_{0}}^{t_{1}}AR_{i,t}$$

The Bucharest Stock Exchange indices analyzed in this paper are BET, BET-TR, BET-FI, BETPLUS, and BET-NG, data being extracted from investing.com and bvb.ro. The reason for choosing them considers their definition and composition. BET is the main index of the Bucharest Stock Exchange, with companies from multiple sectors, with BET-TR reflecting the dividends paid by the constituents of the main index BET. BET-FI and BET-NG are sectorial indices, the first one considering only financial investment firms or other assimilated entities and the second one is an index for energy companies (electricity, oil, and transportation of energy). BETPLUS is a free float market capitalization weighted index of Romanian firms (except for financial investment companies). Its constituents are the most liquid in terms of trading and are from multiple economic sectors. Except for BET-TR, which is adjusted for dividends, all indices reflect changes in market prices.

Analyzing these different indices can shed new light regarding the impact of the COVID-19 on an emerging stock market. For the market return, the S&P Europe 350 Index was considered as a good indicator, which is a measure of the European market that tracks European stocks for the broad market.

In order to analyze different sectors, we used a list of 56 stocks listed on the Bucharest Stock Exchange on the regulated market section. The dataset was initially collected for 83 companies from the Bucharest Stock Exchange, from the regulated market and main exchange segment. Twenty-sevencompanies were removed for having few trading days in the sample period. The daily price to calculate the return for each of the 56 companies was extracted from investing.com (an open-source data website with real-time stock and index information). A validation process was conducted to compare the data from investing.com, using yahoo finance and bvb.ro to eliminate errors. The companies were then arranged using their main activity in different equity sectors (see Appendix A). The Fama and French three-factor model (Fama & French, 1993) was used to estimate the stock abnormal returns. The linear regression used is the following:

(6) $$R_{i,t}=\alpha_{i}+\beta_{i1}\left(R_{m,t}-R_{f,t}\right)+\beta_{i2}\mathrm{SM}B_{t}+\beta_{i3}\mathrm{HM}L_{t}+\varepsilon_{i,t}$$

where *R_i_*,_*t*_ is the actual return of the Romanian stock *i* on day *t*, *R_i_*,_*t*_ is the risk-free return on day *t* using the Romanian 10-Year Bond Yield, *R*_m,*t*_ is the market return for the S&P Europe 350 Index on day *t*, SMB_*t*_ is the difference in returns between small and large stock firms on day *t*, HML_*t*_ is the difference in returns between high and low book-to-market ratio stocks on day *t*, α_*i*_ is the intercept of the relationship for Romanian stock *i*, and ε_*i,t*_ is the error term for Romanian stock *i* on day *t*.

Using an OLS regression, the parameters $\hat{\alpha }$_*i*_, $\hat{\beta }$_*i*1_, $\hat{\beta }$_*i*2_, $\hat{\beta }$_*i*3_ were calculated using an estimation period from thirrd of January 2019 and ending 11 days before the event day, using the same three event days as for the indices. The abnormal return for stock *i* on day *t* and the cumulative abnormal return during the estimation window *t*_0_–*t*_1_ are:

(7) $$AR_{i,t}=R_{i,t}-({\hat{\alpha }}_{i}+{\hat{\beta }}_{i1}\left(R_{m,t}-R_{f,t}\right)+{\hat{\beta }}_{i2}\mathrm{SM}B_{t}+{\hat{\beta }}_{i3}\mathrm{HM}L_{t})$$

(8) $$\mathrm{CA}R_{i}\left(t_{0},t_{1}\right)=\sum_{t=t_{0}}^{t_{1}}AR_{i,t}$$

The average abnormal returns (AARs) for sectors of activity and the cumulative average abnormal returns (CAARs) during the estimation window *t*_0_–*t*_1_ are:

(9) $$\mathrm{AA}R_{t}=\sum_{t=t_{0}}^{t_{1}}\frac{AR_{i,t}}{N}$$

(10) $$\mathrm{CAA}R_{i}\left(t_{0},t_{1}\right)=\sum_{t=t_{0}}^{t_{1}}\mathrm{AA}R_{t}$$

*N* is the number of companies for each type of sector.

In this empirical study, the data was collected from investing.com for daily closing prices for indices and firms and for the Romanian 10-Year Bond Yield, bvb.ro was used to extract the information regarding the activity of each company. Fama and French three factors were taken from the website of Kenneth R. French. To check if the series used in our empirical study are stationary, we have conducted the Augmented Dickey Fuller (ADF) test for unit root. All the 53 stocks used in the analysis and the indices are stationary, the *p*-value of the ADF test was .00 (less than .05), rejecting the null hypothesis of a unit root.

## Empirical Results

### Results for the Romanian Indices

**Figure 1.**
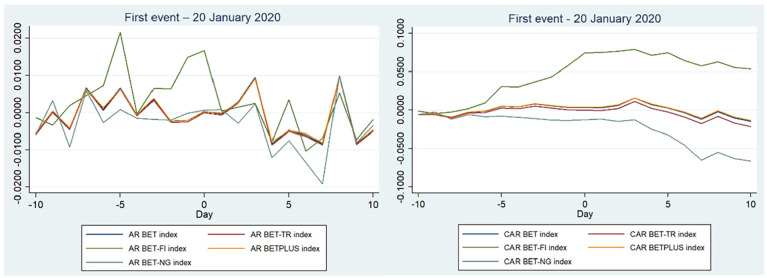
ARs and CARs related to the first event—20 January 2020 for Romanian stock indices.

We start by presenting the graphical illustration (Figure 1) of the overall results related to the ARs and CARs, using the market model as stated above, for the first event, with the start point on the graph on day −10.

Figure 1 illustrates that the concerns of the Chinese media and authorities before and around 20 January 2020 did not have a significant impact on the Romanian stock market, evident from the fact that the CARs are positive for the event window (−10, 0), on the event day (0, 0) and the trend is moving upwards. Believing that the virus is a faraway concern, the companies and authorities did not take any measures to combat the pandemic. This is contrary to our first hypothesis H1a (this hypothesis is not confirmed) and shows that the pandemic was not significant in influencing the stock market in Romania. Tables 1 and 2 highlight that few ARs or CARs were statistically significant. On the event day, most of the indices were positive (BET-FI had a significant positive percentage of +1.66%), starting a small decline to day +10 of the event window. BET-NG (the energy index) was the only index that had the biggest fall after 20 January 2020. This is in line with the reports of the International Energy Agency showing a decrease between 10% and 30% of electricity demand in Europe, which impacted electricity companies the most (IEA, 2020). BET-TR, which tracks the main companies of the BET index, considering also their dividends, has had a big decline in the event window (0, 10), signaling a concern related to dividend pay-outs for investors.

**Table 1.** The Abnormal Returns for the Impact of the First Event (20 January 2020) for Romanian Indices.

Day	BET index		BET-TR index		BET-FI index		BETPLUS index		BET-NG index	
	AR (%)	*t*-Value	AR (%)	*t*-Value	AR (%)	*t*-Value	AR (%)	*t*-Value	AR (%)	*t*-Value
−10	−0.56	−0.67	−0.59	−0.68	−0.14	−0.12	−0.54	−0.66	−0.58	−0.63
−9	0.04	0.24	0.01	0.24	−0.34	−0.23	0.04	0.24	0.32	0.50
−8	−0.43	−0.34	−0.46	−0.35	0.19	0.43	−0.41	−0.34	−0.93	−0.88
−7	0.66	0.99	0.63	1.00	0.45	0.80	0.63	0.97	0.59	0.82
−6	0.09	0.16	0.05	0.16	0.72	1.04	0.13	0.21	−0.28	−0.25
−5	0.66	0.84	0.63	0.85	2.16***	2.89	0.64	0.84	0.08	0.14
−4	−0.05	0.14	−0.09	0.14	−0.03	0.17	−0.05	0.14	−0.15	−0.01
−3	0.36	0.55	0.33	0.55	0.65	0.99	0.38	0.59	−0.19	−0.10
−2	−0.23	−0.08	−0.27	−0.09	0.64	1.03	−0.24	−0.10	−0.21	−0.07
−1	−0.22	0.15	−0.25	0.15	1.48*	2.28	−0.21	0.16	−0.02	0.30
0	0.03	0.09	−0.01	0.09	1.66**	2.25	0.02	0.09	0.06	0.12
1	−0.04	0.01	−0.07	0.01	0.04	0.16	−0.01	0.05	0.08	0.14
2	0.29	0.42	0.25	0.42	0.15	0.31	0.27	0.41	−0.29	−0.25
3	0.95	1.03	0.92	1.04	0.25	0.30	0.92	1.01	0.23	0.17
4	−0.84	−1.33	−0.87	−1.35	−0.77	−1.19	−0.78	−1.30	−1.21	−1.59
5	−0.47	−0.17	−0.51	−0.17	0.35	0.81	−0.48	−0.19	−0.75	−0.50
6	−0.61	−0.48	−0.64	−0.48	−1.03	−1.08	−0.56	−0.44	−1.34	−1.27
7	−0.84	−1.20	−0.87	−1.21	−0.69	−0.98	−0.82	−1.21	−1.93**	−2.26
8	0.99	0.93	0.96	0.94	0.53	0.55	0.98	0.95	0.99	0.90
9	−0.83	−0.83	−0.86	−0.84	−0.73	−0.76	−0.81	−0.83	−0.80	−0.75
10	−0.46	0.09	−0.50	0.09	−0.19	0.30	−0.46	0.08	−0.35	0.12

*Note*. This table reports the daily abnormal returns from equation (4) for the Bucharest Stock Exchange indices used for the first event, with 10 days before and after the event. AR = abnormal return; *t*-value = the value of the *t*-test for significance; BET = Bucharest Exchange Trading; BET-TR = Bucharest Exchange Trading Total Return Index; BET-FI = Bucharest Exchange Trading—Investment Funds; BETPLUS = Bucharest Exchange Trading Plus Index; BET-NG = Bucharest Exchange Trading Energy & Related Utilities Index.

* , **, and *** denote statistical significance at the 10%, 5%, and 1% levels, respectively.

**Table 2.** The Cumulative Abnormal Returns for the Impact of the First Event (20 January 2020) for Romanian Indices.

Event window	BET index		BET-TR index		BET-FI index		BETPLUS index		BET-NG index	
	CAR (%)	*p*-Value	CAR (%)	*p*-Value	CAR (%)	*p*-Value	CAR (%)	*p*-Value	CAR (%)	*p*-Value
(−10, 0)	0.35	.901	−0.01	.997	7.45***	.004	0.39	.888	−1.30	.674
(−5, 0)	0.55	.793	0.35	.866	6.55***	.001	0.54	.789	−0.43	.848
(0, 0)	0.03	.975	−0.01	.995	1.66**	.032	0.02	.978	0.06	.948
(0, 5)	−0.08	.968	−0.28	.894	1.67	.385	−0.06	.977	−1.87	.410
(0, 10)	−1.83	.522	−2.18	.440	−0.44	.868	−1.73	.532	−5.30*	.087

*Note*. This table reports the cumulative abnormal returns from equation (5) for the Bucharest Stock Exchange indices used for the first event, with different time intervals around the event of day 0. CAR = cumulative abnormal return; *t*-value = the value of the *t*-test for significance; BET = Bucharest Exchange Trading; BET-TR = Bucharest Exchange Trading Total Return Index; BET-FI = Bucharest Exchange Trading—Investment Funds; BETPLUS = Bucharest Exchange Trading Plus Index; BET-NG = Bucharest Exchange Trading Energy & Related Utilities Index.

* , **, and *** denote statistical significance at the 10%, 5%, and 1% levels, respectively.

**Figure 2.**
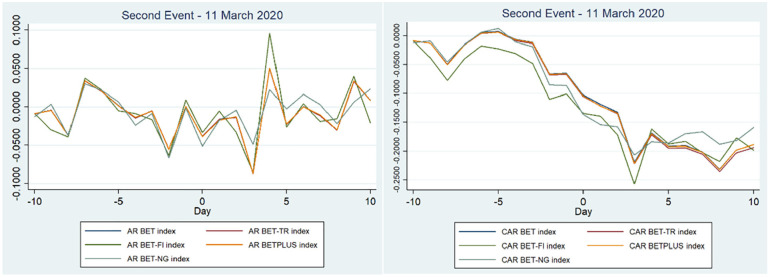
ARs and CARs related to the second event—11 March 2020 for Romanian stock indices.

Considering the second event, 11 March 2020, the situation is different compared to the first event. In the pre-event window (−10, −1) and in the post-event period (1, 10) there was a significant decline in the market, as shown by Figure 2. Investors started to take notice of the situation in China, the virus starting to infect more and more people in Romania and around Europe. Day −10 coincides with the first case of COVID-19 in Romania in which the market reacted negatively.

Tables 3 and 4 show that the pandemic declaration was significant and had a negative impact on the Bucharest Stock Exchange (the percentages on the event day (0, 0) were between −3.36% for BET-FI and −5.09% for BET-NG). This is in line with the literature, which showed that the main index (US index) was a net receiver of shocks during the outbreak of the pandemic in comparison with other assets (Bouri, Cepni, et al., 2021). We can also notice that days −10, −5, and −1 were not statistically significant for all indices; nevertheless, this does not affect our findings concerning the negative impact of the pandemic. Furthermore, there is a noticeable dip in abnormal and cumulative returns on day 3, which is the day of 16 March 2020, the first trading day after the Romanian lockdown was instituted (noticeable in Figure 2). On this day, four indices had the biggest reaction and a considerable negative impact (BET, BET-FI, BET-TR, and BETPlus falling by more than 8.5%), with BET-NG (the energy index) not having the biggest fall, linked with the reversal of energy consumption after the initial fall in the first quarter of 2020 (IEA, 2020). BET, BET-TR, and BETPLUS indices were the most affected by the pandemic with the indices that measure financial investment firms or other assimilated entities and the energy sector being less impacted. BET-TR fell more than the BET index, which shows that investors started to consider the possibility that their dividends would be affected by the pandemic because of the reduction in future profits. The results of the second event have confirmed hypothesis H2a, that the announcement of the pandemic by the WHO had a negative impact on the Romanian stock market.

**Table 3.** The Abnormal Returns for the Impact of the Second Event (11 March 2020) for Romanian Indices.

Day	BET index		BET-TR index		BET-FI index		BETPLUS index		BET-NG index	
	AR (%)	*t*-Value	AR (%)	*t*-Value	AR (%)	*t*-Value	AR (%)	*t*-Value	AR (%)	*t*-Value
−10	−0.84	−0.94	−0.87	−0.95	−0.85	−0.95	−0.84	−0.96	−1.24	−1.33
−9	−0.4*	−1.82	−0.42	−1.83	−2.99***	−4.92	−0.45*	−1.92	0.37	−0.84
−8	−3.68***	−5.81	−3.69***	−5.87	−3.89***	−6.07	−3.71***	−6.01	−3.7***	−5.35
−7	3.43***	4.39	3.4***	4.43	3.75***	5.08	3.43***	4.52	3.02***	3.49
−6	2.06***	3.09	2.02***	3.12	2.23***	3.44	2.05***	3.16	2.17***	2.90
−5	0.21	0.93	0.17	0.94	−0.48	0.01	0.18	0.91	0.68	1.34
−4	−1.41**	−2.19	−1.43**	−2.22	−0.85	−1.42	−1.32**	−2.14	−2.36***	−3.09
−3	−0.47*	−1.93	−0.48*	−1.94	−1.67***	−3.24	−0.52**	−2.03	−0.83**	−2.20
−2	−5.49***	−9.63	−5.49***	−9.72	−6.27***	−10.50	−5.5***	−9.88	−6.62***	−10.08
−1	0.08	−0.17	0.05	−0.17	0.93	1.05	0.09	−0.15	−0.12	−0.41
0	−3.82***	−4.90	−3.84***	−4.95	−3.36***	−4.46	−3.87***	−5.11	−5.09***	−5.91
1	−1.57***	−6.50	−1.56***	−6.56	−0.51***	−4.45	−1.74***	−6.84	−1.72***	−6.15
2	−1.34	−1.05	−1.37	−1.06	−3.28***	−3.69	−1.24	−0.97	−0.39	0.07
3	−8.69***	−12.39	−8.7***	−12.51	−8.5***	−12.40	−8.67***	−12.71	−4.86***	−7.03
4	5.03***	7.29	4.99***	7.36	9.58***	13.43	5.08***	7.55	2.27***	3.52
5	−2.28***	−4.46	−2.28***	−4.49	−2.63***	−4.75	−2.22***	−4.50	−0.24*	−1.83
6	0.09	1.54	0.05	1.56	0.43*	1.84	0.04	1.50	1.63***	3.10
7	−1.08	−0.62	−1.12	−0.62	−1.89*	−1.78	−0.99	−0.54	0.31	0.97
8	−3.02***	−5.28	−3.03***	−5.33	−1.53***	−3.24	−3***	−5.39	−2.18***	−3.91
9	3.38***	7.35	3.32***	7.42	3.99***	7.95	3.42***	7.58	0.64***	3.62
10	0.89**	2.43	0.85**	2.45	−2.07	−1.49	0.84**	2.42	2.33***	3.79

*Note*. This table reports the daily abnormal returns from equaiton (4) for the Bucharest Stock Exchange indices used for the second event, with 10 days before and after the event. AR = abnormal return; *t*-value = the value of the *t*-test for significance; BET = Bucharest Exchange Trading; BET-TR = Bucharest Exchange Trading Total Return Index; BET-FI = Bucharest Exchange Trading—Investment Funds; BETPLUS = Bucharest Exchange Trading Plus Index; BET-NG = Bucharest Exchange Trading Energy & Related Utilities Index.

* , **, and *** denote statistical significance at the 10%, 5%, and 1% levels, respectively.

**Table 4.** The Cumulative Abnormal Returns for the Impact of the Second Event (11 March 2020) for Romanian Indices.

Event window	BET index		BET-TR index		BET-FI index		BETPLUS index		BET-NG index	
	CAR (%)	*p*-Value	CAR (%)	*p*-Value	CAR (%)	*p*-Value	CAR (%)	*p*-Value	CAR (%)	*p*-Value
(−10, 0)	−10.33***	.001	−10.58***	.000	−13.46***	.000	−10.46***	.000	−13.72***	.000
(−5, 0)	−10.89***	.000	−11.02***	.000	−11.71***	.000	−10.93***	.000	−14.34***	.000
(0, 0)	−3.82***	.000	−3.84***	.000	−3.36***	.000	−3.87***	.000	−5.09***	.000
(0, 5)	−12.66***	.000	−12.76***	.000	−8.7***	.000	−12.67***	.000	−10.03***	.000
(0, 10)	−12.41***	.000	−12.69***	.000	−9.77***	.000	−12.36***	.000	−7.28**	.018

*Note*. This table reports the cumulative abnormal returns from equation (5) for the Bucharest Stock Exchange indices used for the second event, with different time intervals around the event of day 0. CAR = cumulative abnormal return; *t*-value = the value of the *t*-test for significance; BET = Bucharest Exchange Trading; BET-TR = Bucharest Exchange Trading Total Return Index; BET-FI = Bucharest Exchange Trading—Investment Funds; BETPLUS = Bucharest Exchange Trading Plus Index; BET-NG = Bucharest Exchange Trading Energy & Related Utilities Index.

* , **, and *** denote statistical significance at the 10%, 5%, and 1% levels, respectively.

**Figure 3.**
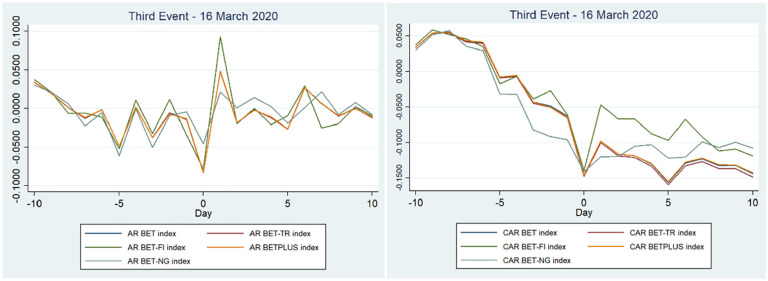
ARs and CARs related to the third event—16 March 2020 for Romanian stock indices.

The beginning of the lockdown in Romania had the biggest impact on the stock exchange compared to the other two events. As mentioned before the drop for the second event was between 3.4% and 6% on the event day (0, 0), compared to an almost double decline for the third event. This was visible on Monday 16 March 2020 when the stock market opened, with a steep decline shown on day 0 (Figure 3). Except for the energy index, which saw a 4.53% drop, all indices fell by more than 7% or 8%.

According to Tables 5 and 6, the CARs for (−10, 0) and (−5, 0) were lower than in the second event, showing persistent negative sentiments regarding the outbreak of the pandemic in Romania. On Day 1 and Day 6, the market reacted positively pushing prices up by more than 2% to 4% (even up by 9% for the BET-FI index), but started to decline after day 2 to day 5 and day 7 to day 10. If we compare the CARs in the post-event window (0, 5) and (0, 10) we can conclude that the negative impact after the declaration of the lockdown was smaller compared to the ones in the second event, nevertheless the impact was still negative (e.g., the CARs range between −1.2% and −9.5% for the third event, but for the second event the range was between −7.3% to a low of −12.7%). Regarding hypothesis H3a, the results show that the lockdown had a negative impact on Bucharest Stock Exchange indices, with a sharp fall on the event day, which disproves our hypothesis. An important point to note is that we do not see a quick reversal occurring in the short time after the event, in comparison with the literature, which saw a reversal after the lockdown (Anh & Gan, 2020; Huo & Qiu, 2020; Ngo & Nguyen, 2021).

**Table 5.** The Abnormal Returns for the Impact of the Third Event (16 March 2020) for Romanian Indices.

Day	BET index		BET-TR index		BET-FI index		BETPLUS index		BET-NG index	
	AR (%)	*t*-Value	AR (%)	*t*-Value	AR (%)	*t*-Value	AR (%)	*t*-Value	AR (%)	*t*-Value
−10	3.43***	4.264	3.4***	4.301	3.76***	4.816	3.44***	4.381	3.02***	3.401
−9	1.98***	3.004	1.95***	3.030	2.1***	3.260	1.97***	3.062	2.11***	2.830
−8	0.11	0.905	0.07	0.912	−0.66	0.013	0.07	0.883	0.60	1.307
−7	−1.27**	−2.133	−1.29**	−2.151	−0.62	−1.347	−1.18**	−2.071	−2.24***	−3.016
−6	−0.14*	−1.871	−0.16*	−1.887	−1.12***	−3.069	−0.19**	−1.967	−0.56**	−2.149
−5	−4.84***	−9.357	−4.84***	−9.437	−5.19***	−9.951	−4.84***	−9.581	−6.1***	−9.822
−4	0.17	−0.162	0.15	−0.164	1.08	0.997	0.19	−0.141	−0.04	−0.401
−3	−3.73***	−4.763	−3.76***	−4.803	−3.22***	−4.227	−3.79***	−4.956	−5.03***	−5.762
−2	−0.58***	−6.318	−0.56***	−6.372	1.18***	−4.213	−0.72***	−6.629	−0.91***	−5.997
−1	−1.42	−1.024	−1.45	−1.033	−3.43***	−3.496	−1.32	−0.939	−0.45	0.069
0	−8.29***	−12.045	−8.3***	−12.148	−7.83***	−11.748	−8.27***	−12.325	−4.53***	−6.854
1	4.83***	7.087	4.79***	7.147	9.25***	12.729	4.88***	7.321	2.12***	3.431
2	−1.89***	−4.335	−1.89***	−4.355	−1.98***	−4.498	−1.83***	−4.364	0.07*	−1.788
3	−0.17	1.499	−0.21	1.512	−0.01*	1.741	−0.22	1.454	1.43***	3.020
4	−1.19	−0.600	−1.22	−0.606	−2.08*	−1.689	−1.11	−0.520	0.23	0.943
5	−2.65***	−5.133	−2.66***	−5.178	−0.91***	−3.068	−2.62***	−5.221	−1.88***	−3.812
6	2.75***	7.140	2.69***	7.201	2.93***	7.532	2.78***	7.345	0.14***	3.526
7	0.65**	2.358	0.61**	2.378	−2.48	−1.410	0.6**	2.346	2.14***	3.695
8	−0.96	0.027	−1.00	0.027	−1.96	−1.183	−0.92	0.070	−0.80	0.136
9	0.03	−1.481	0.02	−1.492	0.25	−1.198	−0.05	−1.609	0.75	−0.560
10	−1.18	−0.729	−1.21	−0.735	−0.97	−0.468	−1.11	−0.664	−0.82	−0.318

*Note*. This table reports the daily abnormal returns from equation (4) for the Bucharest Stock Exchange indices used for the third event, with 10 days before and after the event. AR = abnormal return; *t*-value = the value of the *t*-test for significance; BET = Bucharest Exchange Trading; BET-TR = Bucharest Exchange Trading Total Return Index; BET-FI = Bucharest Exchange Trading—Investment Funds; BETPLUS = Bucharest Exchange Trading Plus Index; BET-NG = Bucharest Exchange Trading Energy & Related Utilities Index.

* , **, and *** denote statistical significance at the 10%, 5%, and 1% levels, respectively.

**Table 6.** The Cumulative Abnormal Returns for the Impact of the Third Event (16 March 2020) for Romanian Indices.

Event window	BET index		BET-TR index		BET-FI index		BETPLUS index		BET-NG index	
	CAR (%)	*p*-Value	CAR (%)	*p*-Value	CAR (%)	*p*-Value	CAR (%)	*p*-Value	CAR (%)	*p*-Value
(−10, 0)	−14.59***	0.000	−14.79***	0.000	−13.94***	0.000	−14.64***	0.000	−14.13***	0.000
(−5, 0)	−18.69***	0.000	−18.76***	0.000	−17.39***	0.000	−18.75***	0.000	−17.06***	0.000
(0, 0)	−8.29***	0.000	−8.3***	0.000	−7.83***	0.000	−8.27***	0.000	−4.53***	0.000
(0, 5)	−9.35***	0.000	−9.49***	0.000	−3.55*	0.082	−9.17***	0.000	−2.57	0.267
(0, 10)	−8.07***	0.005	−8.39***	0.003	−5.78**	0.036	−7.87***	0.005	−1.16	0.710

*Note*. This table reports the cumulative abnormal returns from equation (5) for the Bucharest Stock Exchange indices used for the third event, with different time intervals around the event of day 0. CAR = cumulative abnormal return; *t*-value = the value of the *t*-test for significance; BET = Bucharest Exchange Trading; BET-TR = Bucharest Exchange Trading Total Return Index; BET-FI = Bucharest Exchange Trading—Investment Funds; BETPLUS = Bucharest Exchange Trading Plus Index; BET-NG = Bucharest Exchange Trading Energy & Related Utilities Index.

* , **, and *** denote statistical significance at the 10%, 5%, and 1% levels, respectively.

### Results for the Romanian Stock Market Sectors

Using the Fama and French three-factor model, the impact of the COVID-19 on different sectors was determined. Table 7 related to the first event illustrates that most of the CAARs are not statistically significant. As a result, there was not a negative impact on the event day for almost half of the firms and for the others the decline was in part minimal. This is in line with the results for the indices, not confirming hypothesis H1b, so the announcement of a new virus did not have a negative consequence for the different sectors of the Bucharest Stock Exchange.

**Table 7.** The Cumulative Average Abnormal Returns for the First Event (20 January 2020) for Romanian Stocks by Activity.

	CAAR (−10, 0) (%)	CAAR (−5, 0) (%)	CAAR (0, 0) (%)	CAAR (0, 5) (%)	CAAR (0, 10) (%)
Crude oil and natural gas and services	1.28	1.54	−0.15	−2.22	−4.55
Manufacture of beverages	1.80	1.36	−0.14	−0.06	1.22
Manufacture of paper and paper products	2.97	1.66	−0.04	4.44	2.72
Pharmaceutical and biotechnology	1.52	0.65	−0.55	−1.68	−2.27
Manufacture of rubber and plastic products	8.38**	9.06***	1.77	3.99	3.37
Manufacture of other non-metallic mineral products	1.24	−0.69	2.65*	−2.17	−0.68
Metallurgical industry	0.95	0.13	−0.38	3.40	−1.01
Manufacture of electrical and optical equipment	−0.17	−1.51	2.7**	−1.33	−0.83
Manufacture of machinery and equipment	−0.71	0.12	−0.86	−2.03	−2.46
Electricity production, transportation, and distribution	1.46	1.08	−0.66	−1.57	−3.02
Construction	−5.49	−1.38	−0.68	−3.58	−6.89
Wholesale of metals and metal ores	3.42	2.16	1.90	3.15	2.74
Transportation and storage	−3.53	−2.09	0.05	−0.82	−3.83
Lodging and catering	0.25	1.29	0.77	−0.56	−2.76
Information and communications	2.46	1.29	−0.62	0.57	−1.50
Banking	−2.61	−1.30	−0.71	−0.77	−2.01
Non-banking	5.61**	5.36***	1.47**	1.06	−1.47
Real estate	4.50	3.46	−0.27	−3.32	−2.85

*Note*. This table reports the cumulative average abnormal returns from equation (10) for the economic sectors of activity for the first event. CAAR = cumulative average abnormal return.

* , **, and *** denote statistical significance at the 10%, 5%, and 1% levels, respectively.

The results for the CAARs in the event window (0, 5) and (0, 10) presented in Table 7 show that the oil and gas, pharmaceutical and biotechnology, manufacture of machinery and equipment, electricity, construction, transportation, and real estate sectors were the most affected by the pandemic by day 10, ranging between −1.7% and −6.8%, mentioning that they were not statistically significant. The negative decline is in line with the results found in the literature (Huo & Qiu, 2020; Mukanjari & Sterner, 2020). The decrease in mobility and the absence of industrial activity due to the outbreak of the pandemic significantly decreased the demand for industry material, oil, and other commodities, causing for example the price of oil to sharply decrease, with fears of a global recession (Bouri, Cepni, et al., 2021).

The rapid spread of the virus in Romania and the news of the declaration of it as a pandemic by 11 March 2020 caused a significant impact on the stock market. The CAARs before 11 March in Table 8 were significantly different than after the event day, which implies that there was already panic related to the spread of the virus preannouncement of the WHO. The most affected sectors post event were manufacturing of beverages, metallurgical industry, manufacturing of machinery and equipment, transportation and storage, lodging and catering, banking and real estate activities with CAARs lower than −10%, in line with He et al. (2020) and Liu et al. (2020) findings. Shahzad, Naeem, et al. (2021) have also found that energy, banks, industrials, and consumer discretionary experienced sharp declines in China, in line with our results. An important aspect is that the pharmaceutical and biotechnology sector has negative CAARs, which is in contrast with other findings. This is because of the small health industry in Romania and reliance on medical imports. Most of the CAARs are significantly different from zero at the 1% level. Also, the values of the event window (0, 5) are noticeably lower because the lockdown was on 16 March 2020 which had a consequence on the ARs of the stocks. With social distancing and slowing down of economy activity, the first sectors to be affected were those related to transportation, hospitality, banking, and heavy industry. In conclusion, hypothesis H2b is confirmed, so the announcement of the pandemic had a negative effect on listed companies and sectors on the Bucharest Stock Exchange.

**Table 8.** The Cumulative Abnormal Returns for the Second Event (11 March 2020) for Romanian Stocks by Activity.

	CAAR (−10, 0) (%)	CAAR (−5, 0) (%)	CAAR (0, 0) (%)	CAAR (0, 5) (%)	CAAR (0, 10) (%)
Crude oil and natural gas and services	−21.52***	−17.81***	−7.46***	−11.48***	−10.16***
Manufacture of beverages	−20.52***	−12.41***	−5.02***	−16.99***	−17.61***
Manufacture of paper and paper products	−10.88**	−13.5***	−5.65***	−7.95**	−0.92
Pharmaceutical and biotechnology	−15.16***	−11.56***	−2.38***	−10.82***	−5.23*
Manufacture of rubber and plastic products	−18.3***	−17.64***	−7.2***	−7.54***	−3.93
Manufacture of other non-metallic mineral products	−12.42**	−14.19***	−1.94	−5.2	7.37
Metallurgical industry	−29.93***	−30.06***	−5.55***	−24.71***	−15.64***
Manufacture of electrical and optical equipment	−21.4***	−15.32***	−5.41***	−1.25	2.15
Manufacture of machinery and equipment	−28.85***	−22.74***	−7.2***	−19.54***	−13.2***
Electricity production, transportation, and distribution	−17.26***	−15.59***	−5.81***	−12.77***	−2.71
Construction	−11.05	−8.4	−3.39	−8.49	−5.02
Wholesale of metals and metal ores	−8.03**	−9.67***	−3.34***	−8.71***	1.51
Transportation and storage	−23.26***	−21.67***	−6.8***	−17.05***	−13.54***
Lodging and catering	−28.36***	−22.07***	−7.58***	−26.99***	−13.32***
Information and communications	−21.1***	−17.91***	−5.22***	−22.77***	−7.65
Banking	−18.88***	−16.86***	−4.19***	−20.85***	−16.71***
Non-banking	−18.03***	−15.94***	−4.12***	−9.16***	−7.12***
Real estate	−6.27	−9.31**	−3.33**	−22.77***	−28.68***

*Note*. This table reports the cumulative average abnormal returns from equaiton (10) for the economic sectors of activity for the second event. CAAR = cumulative average abnormal return.

* , **, and *** denote statistical significance at the 10%, 5%, and 1% levels, respectively.

Table 9 shows that that the CAARs for all the sectors were in the range of −10%, with metallurgical industry, ITC, manufacturing of machinery and equipment having fallen by more than 30%. The lockdown had a negative impact on the stock exchange. Most of the sectors in our sample had negative CAARs on the event day, with manufacturing of beverages, metallurgical industry, manufacturing of machinery and equipment, electricity, wholesale of metals and metal ores, transportation and storage, ITC, lodging and catering, banking and real estate falling by more than 6%. These sectors could not adapt fast enough and they rely on movement of people and working on site not from home for labor (Narayan et al., 2021). The lockdown caused a recession that led to a large decrease in energy prices (crude oil), bad loans, and declining interest rates which affected banks and thus the profit margins and stock prices of these companies (Shahzad, Naeem, et al., 2021). Lodging, catering, and food services saw major employee reductions in the wake of the pandemic, which had an indirect spill-over effect on the sectors (Bouri, Naeem, et al., 2021). So hypothesis H3b can be confirmed, namely the lockdown had a negative effect on listed companies and sectors on the Bucharest Stock Exchange. After the lockdown we can conclude by analyzing the event windows (0, 5) and (0, 10) that there was a reversal, especially for sectors like manufacture of paper and paper products, pharmaceutical and biotechnology, manufacture of electrical and optical equipment, electricity production, transportation and distribution, and information and communications. Healthcare, consumer staples, and ITC are the sectors that had a V-turn because, first of all, the health sector was at the forefront in fighting against the pandemic. IT has been helped by implementing the work (and study) from home. Consumer staples were influenced by the growing demand because of the pandemic (Narayan et al., 2021). Banking, real estate, manufacture of beverages and construction are among the sectors that were negatively affected by the lockdown after 10 days of the event. Banking (part of the financial sector) and real estate are strong interconnected sectors as suggested by Shahzad, Bouri, et al. (2021), which is shown by our results to decline because of the lockdown. After the lockdown, the authorities announced plans to stimulate the economy and offer financial aid for employees and companies. The sectors that saw reversals managed to adapt faster to the new market conditions.

**Table 9.** The Cumulative Abnormal Returns for the Third Event (16 March 2020) for Romanian Stocks by Activity.

	CAAR (−10, 0) (%)	CAAR (−5, 0) (%)	CAAR (0, 0) (%)	CAAR (0, 5) (%)	CAAR (0, 10) (%)
Crude oil and natural gas and services	−19.29***	−22.04***	−3.72***	2.57	3.86
Manufacture of beverages	−21.69***	−17.09***	−7.86***	−7.14*	−8.37*
Manufacture of paper and paper products	−15.99***	−17.26***	−3.27**	4.28	10.05**
Pharmaceutical and biotechnology	−14.79***	−16.31***	−2.3***	0.68	4.9*
Manufacture of rubber and plastic products	−11.03***	−17.44***	−3.07***	2.99	−0.8
Manufacture of other non-metallic mineral products	−5.66	−13.68***	0.92	6.96*	6.93
Metallurgical industry	−44.06***	−41.43***	−9.2***	0.92	−2.61
Manufacture of electrical and optical equipment	−15.91***	−16.2***	−1.44	8.77***	8.94**
Manufacture of machinery and equipment	−33.98***	−35.81***	−6.23***	1.86	2.57
Electricity production, transportation, and distribution	−21.36***	−22.33***	−6.81***	2.25	9.33***
Construction	−12.2	−10.12*	−3.12	−3.26	−4.49
Wholesale of metals and metal ores	−14.71***	−14.96***	−10.76***	2.36	0.09
Transportation and storage	−28.43***	−30.21***	−6.44***	−0.05	2.30
Lodging and catering	−25.03***	−22.02***	−1.59	−0.76	−0.78
Information and communications	−29.01***	−32.48***	−8***	1.7***	6.01***
Banking	−23.82***	−22.88***	−7.29***	−12.48	−13.03
Non-banking	−20.14***	−21.35***	−5.75***	0.53	−0.01
Real estate	−21.43***	−24.39***	−9.27***	−20.26***	−19.18***

*Note*. This table reports the cumulative average abnormal returns from equation (10) for the economic sectors of activity for the third event. CAAR = cumulative average abnormal return.

* , **, and *** denote statistical significance at the 10%, 5%, and 1% levels, respectively.

### Link Between Stock Exchange Indices and Listed Firms by Sectors

In the previous sections, we have shown the negative impact of the pandemic on indices related to one stock exchange, namely the Bucharest Stock Exchange and also the effect on sectors. We go on by analyzing the possible link between indices and sectors, with a focus on energy and finance. We chose to analyze the relation between the energy and financial index and the related sectors because the other indices have multiple companies from very different sectors of the economy. The constituents of the energy index are all part of the crude oil and natural gas and services, electricity production, transportation and distribution, and transportation and storage sectors and the same can be said for BET-FI and the non-banking sector. We use the market model for both indices and sectors in order to have the same model as comparison.

Regarding the link between the energy sector and BET-NG, Table 10 shows that the transportation of fuel and storage was negative before the event day of 20 January 2020, and the crude oil and natural gas and services and electricity production, transportation and distribution sectors fell after the event day, with the fall of electricity consumption (IEA, 2020) and the price of oil starting a downward trend. This relationship between the energy sectors and the index is more in detail explained by the fact that the impact of the transportation and storage sector is not high because of the relative small percentage of the sector in the index (almost 14%). The share in the index of the crude oil and natural gas and services sector is almost 58% and about 28% is the share of electricity production, transportation, and distribution. The link is more noticeable in the second event, with significant *p*-values for the results, with crude oil and natural gas and services and transportation and storage sectors having values lower than the energy index (e.g., for the event day (0, 0) crude oil and natural gas and services fell by −7.14% and transportation and storage by −6.48%, with the index dropping by −5.09%), with electricity production, transportation, and distribution sector having only one or two percentage points below the index. The important negative effect of the crude oil and natural gas and services and transportation and storage sectors is, first of all, influenced by the drop of oil prices from 50 dollars per barrel at the end of January 2020 to almost 25 dollars per barrel in Mid-March 2020 and secondly from the reduction in transportation of goods. For the lockdown, the crude oil and natural gas and services sector had one to two percentage points more than BET-NG index, following an upward trend. The negative effect on the energy index was caused by the electricity production, transportation and distribution and transportation and storage sectors till the day of the announcement of the lockdown.

**Table 10.** Link Between Stock Exchange Indices and Listed Firms By Sectors.

	CAAR (−10, 0) (%)	CAAR (−5, 0) (%)	CAAR (0, 0) (%)	CAAR (0, 5) (%)	CAAR (0, 10) (%)
The cumulative average returns for the first event (20 January 2020) for Romanian indices and sectors					
BET-NG index	−1.30	−0.43	0.06	−1.87	−5.30*
Crude oil and natural gas and services	1.57	1.63	0.14	−1.36	−3.64
Electricity production, transportation, and distribution	1.74	1.16	−0.47	−1.12	−2.48
Transportation and storage	−2.76***	−1.85	0.41	0.14	−2.59
BET-FI index	7.45***	6.55***	1.66**	1.67	−0.44
Non-banking	5.80**	5.35***	1.55**	1.80	−0.53
The cumulative average returns for the second event (11 March 2020) for Romanian indices and sectors					
BET-NG index	−13.72***	−14.34***	−5.09***	−10.03**	−7.28**
Crude oil and natural gas and services	−16.67***	−14.22***	−7.14***	−8.55***	−9.45***
Electricity production, transportation, and distribution	−14.61***	−13.53***	−5.65***	−11.42***	−2.71
Transportation and storage	−18.98***	−18.23***	−6.48***	−15.62***	−13.47***
BET-FI index	−13.46***	−11.71***	−3.36***	−8.70***	−9.77***
Non-banking	−12.07***	−11.71***	−3.81***	−4.08***	−4.85***
The cumulative average returns for the third event (16 March 2020) for Romanian indices and sectors					
BET-NG index	−14.13***	−17.06***	−4.53***	−2.57	−1.16
Crude oil and natural gas and services	−11.51***	−15.52***	−2.88***	−0.33	−0.14
Electricity production, transportation, and distribution	−17.43***	−19.06***	−6.41***	−0.11	6.62***
Transportation and storage	−22.48***	−25.83***	−5.91***	−2.59	−0.49
BET-FI index	−13.94***	−17.39***	−7.83***	−3.55*	−5.78**
Non-banking	−10.57***	−13.07***	−4.28***	0.60	−2.44

*Note*. This table reports the cumulative abnormal returns from equation (5) for BET-NG and BET-FI indices and the cumulative average abnormal returns from equation (10) for sectors related to energy and nonbanking. CAR for an index is equal to the CAAR for the same index, because in equation (9), *N* = 1. CAAR = cumulative average abnormal return; BET-NG = Bucharest Exchange Trading Energy & Related Utilities Index; BET-FI = Bucharest Exchange Trading—Investment Funds.

* , **, and *** denote statistical significance at the 10%, 5%, and 1% levels, respectively.

The analysis of the investment funds index (BET-FI) and the non-banking sector highlights some interesting results. The non-banking sector has all the constituents of BET-FI (FP, SIF1, SIF2, SIF3, SIF4, SIF5) plus three other non-banking companies—BVB, BRK, TBK. This is the reason why the values are not identical for each event window. For the three events, the non-banking sector and the investment funds index followed almost the same trend and the sign and values were similar. Differences were noticeable after the WHO announcement when the index fell by −8.77% (0, 5) and −9.77% (0, 10), but the drop was considerably smaller for the non-banking sector (−4.08% for (0, 5) and −4.85% for (0, 10)). Another difference was seen after the lockdown when the non-banking sector had a three or four percentage points more than the index.

The results of the comparison between the energy sectors and non-banking sector and the energy index and investment funds index show a noticeable link, confirming Hypothesis 4. This is an important contribution related not only to the link between the indices and stock sectors during the COVID-19 pandemic but also during specific events. It can have significant consequences for investors and for the financial market depending on what components/companies the index is composed of. Portfolio rebalancing tools must be used to minimize or circumvent risks associated with volatile returns related to a sector and risk spillovers from a sector to another (Salisu et al., 2021), as many times during the COVID-19 pandemic the index (energy or financial) fare better that the associated sectors. As shown above, the diminishing demand for electricity has an impact not only on that sector but relates to a decline of the energy index as well. These results provide useful information for investors to develop efficient portfolio diversification.

## Conclusion

Using the event study methodology, this paper analyses the impact of COVID-19 on Romanian stock indices and sectors. In order to attain this objective, three different event days during the COVID-19 pandemic were chosen, meant to highlight the fact that the impact of this unique pandemic has not been regular on the indices and sectors. The uncertainty created by COVID-19 resulted in financial markets fluctuations (as shown by Zhang et al., 2020), but these reactions were different (remarked by Clemente-Almendros et al., 2021).

The paper has highlighted some important issues. First, we have shown that the Romanian stock market did not react to the news of a new virus causing health issues in China. The investors were not concerned, showing that they do not realize the extent of globalization and transmission of events on financial markets. As infections started to grow in Europe and in Romania, the Bucharest Stock Exchange was negatively affected by the pandemic. The panic caused by COVID-19 negatively affects stock returns through the updating of market risk premium channel (as shown by authors like Aggarwal et al., 2021). Both after 11 March and after 16 March 2020, the Romanian indices have declined. The cumulative abnormal returns on 11 March were far greater 0 to 5 and 0 to 10 days post event compared with the ones for 16 March 2020 (the first trading day after the Romanian lockdown). On the day of the event, the news of the Romanian lockdown had a greater impact on indices than the news on 11 March 2020 when COVID-19 was declared a pandemic. The indices that were the most affected by the events were BET, BET-TR, and BETPLUS, showing that investors were concerned about the prospects of their dividends and the liquidity of the companies on the stock exchange.

Secondly, the impact of COVID-19 on different sectors was in line with the results for the indices. The announcement of a public health emergency in China on 20 January 2020 did not negatively affect the companies traded on the Bucharest Stock Exchange. After the rapid spread of the virus in Romania, the impact was significant. The most affected sectors by social distancing and slowing down of economy activity were those related to transportation, hospitality, banking, and heavy industry, with CAARs lower than −10% and with metallurgical industry, ITC, manufacturing of machinery and equipment having fallen by more than 30% till the lockdown on 15 March 2020. The restrictive measure of staying at home established across Romania after 15 March 2020 had a negative impact on the stock exchange. Manufacturing of beverages, metallurgical industry, manufacturing of machinery and equipment, electricity, wholesale of metals and metal ores, transportation and storage, ITC, lodging and catering, banking and real estate fell by more than 6%. After the lockdown, there was a reversal for sectors like manufacture of paper and paper products, pharmaceutical and biotechnology, manufacture of electrical and optical equipment, electricity production, transportation and distribution, and information and communications. The sectors that saw reversals managed to adapt faster to the new market conditions. Banking, real estate, manufacture of beverages and construction are among the sectors that were negatively affected by the lockdown after 10 days of the event.

Specific indices and sectors are linked and understanding the correlation between them is important, especially in times of unrest. Lastly, we have noticed that the energy index dropped with the fall of the transportation and storage, electricity production, and the crude oil and natural gas and services sectors. An important consequence of the negative impact of specific sectors is their share in the underlying index. The same link was highlighted by the results of the investment funds index and the non-banking sector, with a similar trend.

The paper indicates investors’ confidence and trust in the government’s decisions during the COVID-19 pandemic, favorable stocks prices during three events in Romania and the important link between indices of a stock exchange and sectors.

As far as we know, this is the first paper to analyze the extent of the pandemic on Stock Exchange Indices related to an individual market and in comparison to stock market sectors and thus obtaining important insights. On one side, our results show the negative or positive results related to specific sectors accordingly to the nature of their activities and how they were affected by the pandemic. On the other hand, we highlight the sometimes-different magnitude between indices and sectors, such as, for example, the energy index and energy sector. This is important both for policymakers that have to take into account the link between indices, sectors and ultimately individual companies, and for institutional and retail investors. Understanding that in times of pandemics certain sectors move more or less in the same way and with the same intensity with the specific indices can influence investment strategies and help in hedging against unprecedented events. Knowing what sectors and indices are more sensitive to uncertainty and volatility, sector and index specific stimulus packages may be implemented. Moreover, when seeking stability in financial markets, policy makers may analyze the appropriateness of suitable interventions or support to reduce uncertainties resulting from high volatile periods such as a pandemic. For investors, our findings are important in portfolio management, for diversification and hedging. They show the need to detect the most stressed sectors in high volatility situations such as a pandemic, and then adapt their portfolios choices to their risk profile and characteristics as investors. Based on the knowledge of how the different sectors and indices are affected in periods of large volatility, investors can implement better suitable and adaptive safe haven strategies which may help them to protect their investments.

## Limitations and Future Research

As future lines of research, different statistical methods could be used in order to reinforce our findings, such as regressions. It would be interesting to extent the current analysis to a larger period to check whether the different sectors and indices recover in the same way. As a limitation, our findings are based on a single country. It would be interesting to compare them with other countries or greater regions, including developed or emerging economies.

## Appendix A: Sectors of Activity for the 56 Firms Listed on the Bucharest Stock Exchange

**Table A1.** The 56 Listed Companies Arranged by Sector of Activity.

Sector of activity	Stock company codes/tickers
Crude oil and natural gas and services	SNP, SNG, PTR, RRC
Manufacture of beverages	WINE
Manufacture of paper and paper products	VNC
Pharmaceutical and biotechnology	ATB, BIO, SCD, M, RMAH, RPH
Manufacture of rubber and plastic products	TRP, ROCE
Manufacture of other non-metallic mineral products	CEON, PREB
Metallurgical industry	ALR
Manufacture of electrical and optical equipment	ELMA, AAG, EPT, ELGS
Manufacture of machinery and equipment	ALT, CMP, SNO, ARS, IARV, TBM
Electricity production, transportation, and distribution	SNN, TEL, EL
Construction	IMP, COMI
Wholesale of metals and metal ores	ALU
Transportation and storage	COTE, TGN, OIL, SOCP
Lodging and catering	BCM, EFO, TUFE
Information and communications	BNET, DIGI
Banking	BRD, EBS, PBK, TLV
Non-Banking	FP, SIF1, SIF2, SIF3, SIF4, SIF5, BVB, BRK, TBK
Real estate	SFG
